# A recommended workflow methodology in the creation of an educational and training application incorporating a digital reconstruction of the cerebral ventricular system and cerebrospinal fluid circulation to aid anatomical understanding

**DOI:** 10.1186/s12880-015-0088-6

**Published:** 2015-10-19

**Authors:** Amy Manson, Matthieu Poyade, Paul Rea

**Affiliations:** Laboratory of Human Anatomy, School of Life Sciences, College of Medical, Veterinary and Life Sciences, University of Glasgow, Glasgow, G12 8QQ UK; Digital Design Studio, Glasgow School of Art, Glasgow, G51 1EA UK

**Keywords:** Ventricular, Neuroanatomy, 3D, Volumetric visualisation, Education

## Abstract

**Background:**

The use of computer-aided learning in education can be advantageous, especially when interactive three-dimensional (3D) models are used to aid learning of complex 3D structures. The anatomy of the ventricular system of the brain is difficult to fully understand as it is seldom seen in 3D, as is the flow of cerebrospinal fluid (CSF). This article outlines a workflow for the creation of an interactive training tool for the cerebral ventricular system, an educationally challenging area of anatomy. This outline is based on the use of widely available computer software packages.

**Methods:**

Using MR images of the cerebral ventricular system and several widely available commercial and free software packages, the techniques of 3D modelling, texturing, sculpting, image editing and animations were combined to create a workflow in the creation of an interactive educational and training tool. This was focussed on cerebral ventricular system anatomy, and the flow of cerebrospinal fluid.

**Results:**

We have successfully created a robust methodology by using key software packages in the creation of an interactive education and training tool. This has resulted in an application being developed which details the anatomy of the ventricular system, and flow of cerebrospinal fluid using an anatomically accurate 3D model. In addition to this, our established workflow pattern presented here also shows how tutorials, animations and self-assessment tools can also be embedded into the training application.

**Conclusions:**

Through our creation of an established workflow in the generation of educational and training material for demonstrating cerebral ventricular anatomy and flow of cerebrospinal fluid, it has enormous potential to be adopted into student training in this field. With the digital age advancing rapidly, this has the potential to be used as an innovative tool alongside other methodologies for the training of future healthcare practitioners and scientists. This workflow could be used in the creation of other tools, which could be developed for use not only on desktop and laptop computers but also smartphones, tablets and fully immersive stereoscopic environments. It also could form the basis on which to build surgical simulations enhanced with haptic interaction.

## Background

Prior to modern imaging modalities, visualising structures inside the skull was problematic. Previously, identification of space-occupying lesions within the skull was done using air ventriculography, introduced in 1918 by the American neurosurgeon Walter Dandy. This allowed the identification of tumours of the brain by radiolucent changes of the ventricular system [1]. This procedure was dangerous and replaced in the late 1970s by advances in imaging technology. With the invention of less invasive techniques such as computerised tomography (CT) and magnetic resonance imaging (MRI) scans, the brain and ventricular system could be visualised with much reduced risk to the patient. These techniques greatly improved the quality of neuroimaging, especially with the introduction of T1 and T2 weightings of MRI scans, allowing specific structures to be highlighted. Nowadays, with further advances in computer technology, high quality scans and computer software can be combined to create a three-dimensional (3D) model from a 2D medical imaging dataset. These models can be used diagnostically, and as an aid to teaching, for demonstrating complex or inaccessible anatomy, including that of the ventricular system of the brain. Indeed, the demonstration of the ventricular system is also challenging to demonstrate in the teaching environment, and visualised only as “spaces” within the dissecting room which, once compromised, will never appear the same to the learner. Therefore, to enable clear demonstration of the anatomy of the ventricular system using meaningful datasets would allow a greater understanding of these structures, including the flow of cerebrospinal fluid (CSF), again something which is not able to be demonstrated within the dissecting room.

## 3D models and technology in learning

Traditional learning methods in anatomy focus on the use of textbooks, dissection, 2D illustrations or diagrams and medical imaging techniques. Spatial relations are difficult to appreciate, and this certainly applies to the cerebral ventricular system and the flow of CSF within the brain and spinal cord. Visualising these types of structures in 3D with interactive educational material can greatly improve the understanding of spatial relationships and retention of that knowledge [2, 3]. Marks [4] asserts that, as the human body is a 3D object, anatomical education should involve learning and handling 3D information.

Recent developments in computer software and improvements in medical imaging quality have allowed anatomically accurate 3D models to be created, which can then be used as a computerised 3D model for learning. These educational models tend to be most effective when incorporated in an interactive context such as an interactive learning application, or when used to create animations of anatomical or physiological subjects [5].

The shape of anatomical structures and their spatial relationships is best understood if the user can interact with the created 3D models as they gain a sense of the 3D structure and spatial relationships with surrounding structures not easily understood by other means [2, 3]. With cadaveric specimens this can only be achieved within the dissection laboratory and for other materials, such as bones, there is often limited availability. Prosections (professionally dissected and preserved specimens) may be impractical to maintain and examples of gross pathology may not be available, leaving students to learn solely through textbooks with written descriptions and 2D images. Evidence suggests that students prefer to learn using both 2D images and 3D interactive models, compared to using solely 2D or 3D information [6]. Using computerised 3D models allows the anatomy to be accessed anywhere, and do not rely on access to the dissection room or prosection materials [7]. Many 3D brain and whole body atlases are available on the market for educational purposes (e.g., Anatomy Browser, Zygote Body and BodyParts3D), all of which have been created from MRI or CT scan data. Some, including Anatomy Browser, also have the capability of providing surgical simulations [8].

Computer-based learning applications offer advantages over more traditional learning methods [9–11]. Computerised learning offers students control over content, learning sequence, and pace of working through the educational material. These applications can be paused at any time, and repeated as often as needed with the user learning from their mistakes. Users of this type of material tend to be more motivated than with traditional learning methods as they can progress through them at their own pace, thereby enhancing the learning experience [9–11]. It has been suggested that a computer-based educational programme could be as effective and beneficial as traditional methods, with improved rural and remote access [9–11].

Ruiz et al. [11] suggested that computerised learning should not be seen as replacing traditional instructor-led training but as a complement to it, forming part of a blended-learning strategy, with similar findings shown in a later study by Petersson et al. [12]. This indicates that perhaps interactive computer models would be most beneficial when combined with other teaching methods such as practical dissection.

It has been shown that, while those individuals with high spatial ability tended to learn more effectively than those with low spatial ability when using computer-based 3D anatomy models, if care was taken to include “reference points” for the model, such as orientation references, both groups of individuals achieved a similar level of learning [13]. A study by Brewer at al. [14], focussing on learning brain anatomy, suggests that interactive computer-aided learning may actually be beneficial to those with low spatial awareness as the computer-based learning aided the cognitive leap from 2D textbook images to 3D dissections. They also suggested that this method of learning may be beneficial for individuals new to that area of anatomical learning.

However, the creation of an overly complex computer model can have a negative impact on the learning experience as the user can become overwhelmed by too much information given at once [15, 16]. This is especially true of animated visualisations which are only effective if carefully designed and appropriate to the data (such as conveying change over time or space) [17]. A balance has to be struck between an interactive computer model and the level of detail incorporated into it by minimising unnecessary or extraneous cognitive load (total mental effort used in the working memory). It should be noted that individuals have varying levels of cognitive load [16]. The addition of visualisation to text reduces the load on the working memory, freeing it up for processing of the information, thereby leading to better understanding [18, 19]. Individuals have a limited capacity for processing information - only a small amount of information can be processed at a time. Meaningful learning occurs through active processing where individuals are engaged in thinking about the information given, possibly through solving problems, rather than passively reading or viewing information [20, 21]. However, allowing the user active control of the visualisation was sometimes not as effective as providing an animation highlighting key areas through optimal movements of the visualisation. This was due to differences in the manipulation of the visualisation, which seemed unrelated to spatial awareness. In the study by Keehner et al. [22], individuals who passively viewed optimal movements of the visualisation performed the same in the task as those who actively manipulated the visualisation effectively, and performed better than those who manipulated it ineffectively.

Estevez et al. [23] examined the effectiveness of the use of physical 3D models in relation to the anatomy of brain. They found that those participants who learned using the 3D model performed better in tests than those who learned using the traditional 2D method, especially on questions relating to 3D relations of the surrounding anatomy of specific neural structures [23]. A later study by Pani et al. [24] also concluded that learning of neuroscience and other biomedical subjects was improved by the inclusion of computer-based learning in the curriculum. Virtual computer-based 3D models in neuroanatomical teaching build on and enhance traditional methods of learning: they are easy and cheap to share, but can also allow surgical simulations using haptic interfaces and tactile feedback to be incorporated into the teaching material. In addition, 3D computer models can also be accompanied by animations to demonstrate the flow of fluid (e.g., CSF flow around the ventricular system), something which cannot be easily demonstrated in the dissecting room, or through static images [25].

With the effectiveness of 3D models and computer-based anatomical learning in mind, Adams and Wilson [26] developed a 3D interactive teaching application based on MRI data focussing on one aspect of the brain – the ventricular system. Using this application, students had the option of adding or removing structures, altering opacity and creating cross-sections to view internal structures. It included a video animation of the flow of CSF though the ventricles. The 3D models could be viewed stereoscopically to increase depth perception and to emphasise the spatial relationship of the ventricular system within the surrounding structures and were developed for both self-directed and classroom-based teaching [26].

With the many advantages of 3D imaging and reconstruction, especially applied to the cerebral ventricles and flow of CSF, the purpose of this study was to establish a workflow in the creation of a manageable interactive dataset of the ventricular system and CSF flow aimed at undergraduate students and also to investigate how to create a self-test function on knowledge within this application. This was to be achieved using widely available software packages and would result in a standalone application for use on Windows® and MacOS desktop and laptop computers, with the capability of becoming a solely web-based application.

## Materials and methods

A 3D model of the ventricular system of the brain was generated by highlighting chosen anatomical structures from an MRI dataset using 3D data visualisation and processing software. This model was then edited using 3D computer graphics software and textured using bitmaps created using a raster graphics editor, then exported to a game creation system to create an interactive application. 3D computer graphics software and a compositing programme were used to create two animation videos, which were then imported into the game creation system and combined with background music edited in a digital audio editor. The application was created in ~10 weeks by a single individual working with around 10 additional weeks of “learning time” spent with the software packages.

### Materials

The dataset used in this study was of magnetic resonance (MR) images of a human head, provided by the University of Erlangen-Nuremburg [http://www9.informatik.uni-erlangen.de/External/vollib/]. Ethical approval was not required for the use of the MR data as it is in the public domain. The volumetric dataset (http://www9.informatik.uni-erlangen.de/External/vollib/MRI-Head.pvm) used in this study has been kindly provided by the Computer Graphics Group from the Friedrich-Alexander Universitat Erlangen-Nurnberg (http://lgdv.cs.fau.de/) through the volumetric library webpage (http://www9.informatik.uni-erlangen.de/External/vollib/), exclusively for research with no commercial purposes.

**Fig. 1:**
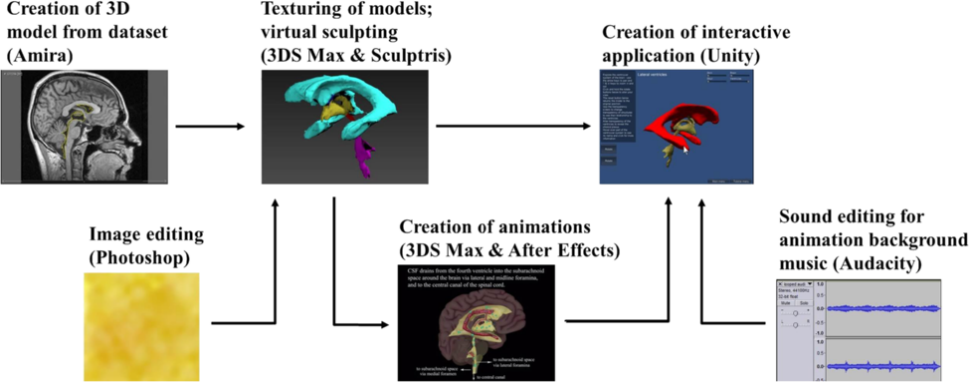
Workflow model proposed in the creation of an educational interactive application with animations using real MRI data of the ventricular system of the brain

The authors state that the work presented in the paper entitled "A Novel Workflow Methodology In The Creation Of An Educational And Training Package To Aid understanding Of The Anatomy Of The Cerebral Ventricles, Incorporating Flow Of Cerebrospinal Fluid" is a research work and has no commercial purposes. In addition, on the website (http://www9.informatik.uni-erlangen.de/External/vollib/), it states unmodified PVM datasets are allowed to be redistributed, and that is the only ones present in Figs. 1 and 3 in that format of MRI. The other images that we have created have been designed in other software packages and are not directly related to the MRI datasets, but only used as guidance. The Figs. 1 and 3 therefore have permission to be used in this context.

Due to their wide availability and relative ease of use, the following software packages were used (Table 1) The interactions between these packages and a plausible workflow are demonstrated in Fig. 1:

**Table 1:** This table shows the computer software and programmes, supplementary 3D models and dataset used within this study and some free alternatives

Computer software	Programmes used	Developing company	Use in this study	Availability	Potential free alternatives
3D data visualisation and processing	Amira 5.4.5	FEI Visualisation Sciences Group; MA, USA	Creation of 3D model from dataset	Institution licence available	3DSlicer
Raster graphics editor	Adobe Photoshop Elements	Adobe; CA, USA	Image editing	Free trial available	ImageJ
3D computer graphics	Autodesk 3DS Max	Autodesk; CA, USA	Texturing of models; creation of animations (using Zygote Body models)	Free trial available	Blender
3D computer graphics	Sculptris	Pixologic; CA, USA	Virtual sculpting	Free open-source	-
Compositing programme	Adobe After Effects CC	Adobe; CA, USA	Creation of animations	Free trial available	DebugMode Wax
Game creation system	Unity3D game engine 4.5.2f1	Unity Technologies; CA, USA	Creation of interactive application (using BodyParts3D models)	Free version available; paid version needed for video playback (~£48/month)	CryEngine (CryTek; Frankfurt, DE) or UnrealEngine (Epic Games; MD, USA)
Digital audio editor	Audacity	-	Sound editing for animation background music	Free open-source	-
	Supplementary 3D models used				
	Zygote Body	Zygote Media Group, 2013	Used in animations	Paid licence^a^	BodyParts3D
	BodyParts3D	Anatomography, 2009	Used in interactive application	Free open-source	-
	Dataset				
	MRI of human head	University of Erlangen-Nuremburg	Creation of 3D cerebral ventricular model	Free open-source	-

^a^Zygote Body model was used in animations in this study as a paid licence was already in place (available for download from [http://www.3dscience.com] and available to view for free at [https://zygotebody.com]). Zygote Body could easily be replaced by BodyParts3D or any other free open-source models

### Methods

Using Amira 5.4.5, a 3D model of the ventricular system of the brain was generated by segmenting chosen anatomical structures from an MRI dataset. This approach has been successfully demonstrated in complex anatomical regions by Nguyen and Wilson [27] and Sergovich et al. [28], and specifically with brain anatomy by Yeung et al. [29] and Pedersen et al. [30]. This model was edited in Autodesk 3DS Max and textured using bitmaps created in Adobe Photoshop Elements, then exported to Unity3D game engine to create an interactive application. 3DS Max and Adobe After Effects were used to create two animation videos, which were then imported into Unity3D and combined with background music edited in Audacity. The study was achieved in ~10 weeks by a single individual working with some prior knowledge (again, ~10 weeks) of the software.

### Segmentation and surface generation

The ventricular system of the brain was isolated and highlighted on each 2D cross-section of the MRI dataset through a combination of semi-automated and manual segmentation in Amira 5.4.5 involving separating voxels (3D pixels) of different intensity values to select certain areas and structures. A combination of tools was used to achieve this: the blow tool (semi-automatic) and paint brush tool (manual). The blow tool uses a region-growing technique to highlight like-coloured areas according to their colour gradient and was especially useful when highlighting areas which contrasted strongly with those around it. The paintbrush tool, used to manually select less contrasted areas of the ventricular system and to refine the previous sections made with the blow tool, works by selecting or deselecting an area when clicked. Segmentation and interpolation of segmented data between axial, sagittal and coronal cross-sectional slices enabled the selection of the main structures of the ventricular system (Table 2), which were segmented separately.

**Table 2:** This shows the main structures of the ventricular system identified during the segmentation process in Amira 5.4.5

Lateral ventricles	
Interventricular foramina	
Third ventricle	
Cerebral aqueduct	
Fourth ventricle	

A series of isosurfaces were generated, wrapping around each of the chosen structures within the cerebral ventricular system to create several 3D models. Isosurface generation incorporated constrained smoothing which improved visual appearance without altering the structures produced during the segmentation process. This was done using the standard “constrained smoothing” algorithm, and was the same for each isosurface generated. It was found that further smoothing in Amira resulted in a “blocky” appearance, so the new models were imported into Sculptris as OBJ files for additional smoothing. Sculptris offered more control over the smoothing process compared to Amira by retaining the geometry of the original data, resulting in a more aesthetically pleasing yet accurate model. The smoothed models were then imported into Autodesk 3DS Max for post-processing and rendering, again, as OBJ files. As each structure of the ventricular system had been identified as a unique structure they could be saved individually from Amira 5.4.5 allowing easier manipulation in Sculptris, Autodesk 3DS Max and Unity3D game engine.

### Post-processing, rendering and animation

A material with an attached bitmap image, was assigned to each object in Autodesk 3DS Max. A material is data that is assigned to the surface of an object to affect the way it appears when rendered e.g., colour, glossiness. Bitmaps are images assigned to materials to give surface texture to the object. In addition, models representing the choroid plexus were created then textured using a bump map, which provides the illusion of relief on flat rendered surfaces by adding highlights and shadows.

The lateral, third and fourth ventricle models were subdivided into smaller structures (Table 3) allowing the smaller structures to be exported separately to Unity3D game engine (as FBX files) to allow individual identification in the interactive application.

**Table 3:** This shows the subdivisions of the anatomical structures in the lateral, third and fourth ventricles which were edited in Autodesk 3DS Max

Original object (Amira)	Subdivided models (3DS Max)
Lateral ventricles	Anterior horn
	Body of lateral ventricles
	Atrium
	Posterior horn
	Inferior horn
Third ventricle	Supraoptic recess
	Infundibular recess
	Suprapineal recess
	Pineal recess
	Third ventricle (body)
Fourth ventricle	Lateral foramina (of Luschka)
	Medial foramen (of Magendie)
	Fourth ventricle (body)
	Central canal

Two animations were created using 3DS Max and After Effects. Within these animations, Zygote Body skin, brain and spinal cord 3D models were used to give the created cerebral ventricular model some context by providing some surrounding structures. The Zygote Body 3D models were provided by the Glasgow School of Art (Digital Design Studio) as FBX files:

- **Introduction animation** – The Zygote Body skin, brain and spinal cord 3D models were imported into 3DS Max and aligned with the created ventricular system model in Autodesk 3DS Max and scaled accordingly. A three-point lighting scheme was added to illuminate the scene and a target camera was set. This camera was attached to a 3D path using Path Constraint, meaning that the camera could move only along the line of the path, thereby creating a smooth camera motion. The models were rotated and keyframes were set, while the camera moved towards the models on its path, giving the models a smooth spinning action while the camera zoomed in.
  The animation was rendered out in layers in Autodesk 3DS Max to reduce the time spent rendering and to make the animation easier to manage in After Effects. The renders were then layered together in After Effects. This also allowed the transparency of some of the models to be adjusted in post-production using opacity keyframes, so that the outer models could be “peeled away” to reveal the cerebral ventricular system. Altering the transparency was performed in After Effects as it was more flexible, easier to control and gave a smoother result than in 3DS Max.
- **CSF flow tutorial animation** – The Zygote Body brain and spinal cord 3D models and the created ventricular system model were used in this animation. The focus of this animation was on the fluid flow within the model, so a static target camera and a free spot light were used. Several small spheres were used to represent CSF and were attached to paths within the ventricular system in Autodesk 3DS Max, limiting their movement. Keyframes were set to control the visibility of the spheres and the timing of their movements through the ventricles, and to give them a smooth movement along their paths. The speed of movement was timed to imitate CSF pulsing within the ventricular system with a heartbeat of 75 beats per minute (resting heart rate).
  The animation was created by rendering i.e., generating 2D images from the 3D models. This was done in several separate layers: the left and right brain models and the ventricular system model were rendered separately as still images, as these had no need to move in the animation; and each animated sphere was individually rendered, as these had to be able to move around each other and the other models independently. The renders were imported into Adobe After Effects, where opacity keyframes were added to allow the brain and part of the ventricular system to fade out smoothly. Masks were used on the CSF “particle” layers to give the appearance that each sphere was passing under part of the lateral ventricle whereas, in fact, the lateral ventricle was a 2D still image. The mask opacity was animated using keyframes to allow each sphere to be visible when it first passed over the area of the lateral ventricle in question, then to mask each sphere itself when it next passed that point, giving the impression that it had passed underneath.
  Additional content in the form of textual information was added at the top of the screen, and each sphere animation was copied multiple times over the course of the entire animation to give the user time to read the text and watch the animation.

Table 4 details the models used within each animation video. Both animations were rendered out as QuickTime video format in After Effects and were added to the interactive application in Unity3D game engine by applying each video to a plane.

**Table 4:** This table demonstrates the models used within each animation video, and the specific anatomical structures used

Animation video	Models used
Introduction	• Ventricular system
	- Lateral ventricles
	- Interventricular foramina
	- Third ventricle
	- Cerebral aqueduct
	- Fourth ventricle
	• Zygote Body
	- Brain and spinal cord
	- Skin
CSF flow	• Ventricular system
	- Lateral ventricles
	- Interventricular foramina
	- Third ventricle
	- Cerebral aqueduct
	- Fourth ventricle
	• Choroid plexus
	• CSF “particles”
	• Zygote Body
	- Brain and spinal cord

### Interactive application

The interactive application was developed as a standalone application using Unity3D game engine. The interactive application contains the created ventricular system model, BodyParts3D models (providing some context to the ventricular system model) and created animations within a graphical user interface created using Unity C# (C Sharp) programming language.

**Fig. 2:**
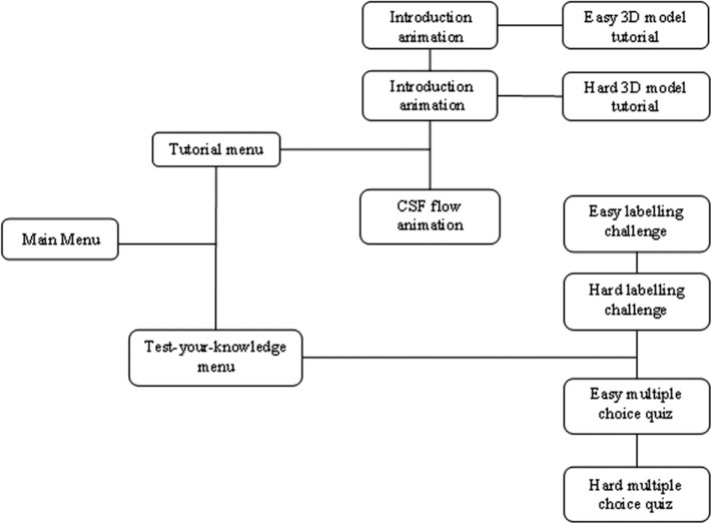
Block diagram of the scene layout created using Unity3D game engine. The application is split into two main parts: a tutorial section with an interactive 3D model of the ventricular system and an educational animation on CSF flow; and a test-your-knowledge area with a labelling challenge and multiple choice quiz at different difficulty levels, allowing the user to test themselves on what they have learned in the tutorial section

The interactive application consists of two main sections: (1) a section including several tutorials and (2) a test-your-knowledge section (Fig. 2). (1) The tutorials comprise two animations; and a labelled 3D interactive ventricular model, where users can pan, zoom and rotate through the use of keyboard keys and on-screen buttons. They can also display the name of areas of the ventricular system, with additional information related to anatomical structures provided by pop-up windows. (2) The test-your-knowledge area proposes two learning challenges – a labelling challenge, in which the user drags and drops labels onto the different parts of the 3D ventricular model, requiring correct identification of the areas of the cerebral ventricular system; and a multiple choice quiz, assessing knowledge of structure and function relating to the areas of the cerebral ventricular system. For both challenges two levels of difficulty are proposed, aiming to enhance self-paced learning. Both difficulty levels would assess knowledge (specifically, memorisation of facts) – level 1 of Bloom’s Taxonomy of Learning [31].

Fig. 2 shows the scenes and menu interfaces created in Unity3D game engine. A main menu interface leading to two sub-menus that enable launching either (1) an introduction animation followed by a 3D model tutorial scene, with two possible levels of complexity; (2) a CSF flow animation tutorial; or (3) a choice of two interactive gaming scenes: (4) a labelling challenge or (5) a quiz, both proposed with two levels of difficulty. All relevant objects imported into the scenes as FBX models from Autodesk 3DS Max are detailed in Table 5, along with two animation videos, imported from Adobe After Effects. Each scene includes a side label explaining the functionality of that level.

**Table 5:** This highlights the models used within each Unity3D scene

Scene	Models used
Introduction animation	• Introduction animation video
Easy 3D model tutorial	• Ventricular system
	- Lateral ventricles
	- Interventricular foramina
	- Third ventricle
	- Cerebral aqueduct
	- Fourth ventricle
	• Choroid plexus
	• BodyParts3D
	- Brain and spinal cord
	- Skull, mandible, and atlas and axis vertebrae
	- Skin
Hard 3D model tutorial	• Ventricular system
	- Anterior horn (lateral ventricle)
	- Body (lateral ventricle)
	- Atrium (lateral ventricle)
	- Posterior horn (lateral ventricle)
	- Inferior horn (lateral ventricle)
	- Interventricular foramina
	- Supraoptic recess (third ventricle)
	- Infundibular recess (third ventricle)
	- Third ventricle
	- Pineal recess (third ventricle)
	- Suprapineal recess (third ventricle)
	- Cerebral aqueduct
	- Fourth ventricle
	- Lateral foramina (fourth ventricle)
	- Medial foramen (fourth ventricle)
	- Central canal (fourth ventricle)
	• Choroid plexus
	• BodyParts3D
	- Brain and spinal cord
	- Skull, mandible, and atlas and axis vertebrae
	- Skin
CSF flow animation	• CSF flow animation video
Easy labelling challenge	• Ventricular system
	- Lateral ventricles
	- Interventricular foramina
	- Third ventricle
	- Cerebral aqueduct
	- Fourth ventricle
Hard labelling challenge	• Ventricular system
	- Anterior horn (lateral ventricle)
	- Body (lateral ventricle)
	- Atrium (lateral ventricle)
	- Posterior horn (lateral ventricle)
	- Inferior horn (lateral ventricle)
	- Interventricular foramina
	- Supraoptic recess (third ventricle)
	- Infundibular recess (third ventricle)
	- Third ventricle
	- Pineal recess (third ventricle)
	- Suprapineal recess (third ventricle)
	- Cerebral aqueduct
	- Fourth ventricle
	- Lateral foramina (fourth ventricle)
	- Medial foramen (fourth ventricle)
	- Central canal (fourth ventricle)
Easy multiple choice quiz	• Ventricular system
	- Lateral ventricles
	- Interventricular foramina
	- Third ventricle
	- Cerebral aqueduct
	- Fourth ventricle
Hard multiple choice quiz	• Ventricular system
	- Anterior horn (lateral ventricle)
	- Body (lateral ventricle)
	- Atrium (lateral ventricle)
	- Posterior horn (lateral ventricle)
	- Inferior horn (lateral ventricle)
	- Interventricular foramina
	- Supraoptic recess (third ventricle)
	- Infundibular recess (third ventricle)
	- Third ventricle
	- Pineal recess (third ventricle)
	- Suprapineal recess (third ventricle)
	- Cerebral aqueduct
	- Fourth ventricle
	- Lateral foramina (fourth ventricle)
	- Medial foramen (fourth ventricle)
	- Central canal (fourth ventricle)

#### Animation scenes

Both animation scenes - introduction animation and CSF flow animation - display, in the first instance, their corresponding animated video. When the introduction animation has been played, the 3D model tutorial scene is loaded automatically. In contrast, the CSF flow animation, and its accompanying background music, can be played and paused by clicking a play/pause button. The background music was edited and looped in Audacity sound editor before being imported into Unity3D game engine. Both animations are in QuickTime video format added to planes, which retains the video’s aspect ratio no matter the size and shape of the user’s computer screen.

#### 3D model tutorial scenes

Two levels of difficulty are proposed: easy and hard. For the easier level of difficulty, the scene was populated with the original ventricular system model, whereas for the harder level of difficulty, the model consisted of a more detailed version including several subdivisions of the original model. In both versions, the user could zoom and pan the camera using keyboard keys. Additionally, two buttons located on the left side of the application window allow rotation of the 3D model around the x and y axes by clicking with the mouse. A reset button enables reloading of the original configuration of the scene.

The opacity of the skin, skull and brain could be adjusted using a horizontal slider, aiming to enhance the understanding of the spatial relationship between the ventricular system and surrounding anatomical structures. The transparency of the ventricular system can also be changed, allowing the location of the choroid plexus to be seen within the ventricles. When the user passes the mouse cursor over an area of the 3D model, it highlights in red and a text label with the name of the region appears. When clicking an area of the model, the corresponding structure is highlighted in blue and a window providing detailed information on that area pops up.

#### Labelling challenge scenes

As previously mentioned, two levels of difficulty are also proposed in the labelling challenge. The easy level uses the 3D model of the ventricular system without subdivision, whereas in the hard level the model is subdivided into relevant parts so that areas from the lateral, third and fourth ventricles can be separately labelled to test for more advanced knowledge. Both difficulty levels include a box containing the labels to be dragged and dropped onto the corresponding part of the ventricular system. Correctly placed labels are displayed in another box, entitled "Solved". On correct labelling, an audio sound is played and a text label appears providing positive reinforcement with the message “Correct!”, then the label in question jumps to the “Solved!” box. On incorrect labelling, a different audio sound is played and a text label appears providing negative feedback with the message “Try Again”. On completion of the labelling game, a text message stating “Well Done!” appears and a congratulatory audio sound is played.

Again, the model is interactive, allowing camera pan, zoom and rotation of the model.

#### Multiple choice quiz scenes

The multiple-choice quiz also offers the choice of two degrees of complexity. Both difficulty levels contain a 3D ventricular system model: the easy level contains the original model whereas the hard level contains the more complex subdivided model. Again, as in the tutorial scene, the view can be panned and zoomed and a set of buttons allows rotation of the model around the x- and y-axes.

An information box located on the left provides the user with the number of questions that are present on the model so they know if there are still more to be found. When the mouse passes over an area of the ventricular system, the model changes colour to red. This allows the user to see the different areas of the model which can be selected. On clicking on an area of the model, a multiple-choice question appears at the top of the screen. Most questions have four possible answers, only one of which is correct. These possible answers have been chosen in such a way that there should be no obviously wrong answer and all could be plausible. On the selection of an answer, the selected area of the ventricular model changes colour to blue-green to mark it as having been already assessed. A correct answer plays an audio sound giving positive reinforcement and a wrong answer plays a different audio sound which provides negative feedback. After answering, the user clicks a “Continue” button to close the answered question and return to the model in order to select another area.

The score is displayed at all times on the screen. At the end of the quiz, the score is provided as fraction of the number of questions. A congratulatory message, or a message advising the user to revisit the tutorial section of the application and then attempt the quiz again, is also displayed, dependent on the final score.

## Results

### Segmentation and surface generation

**Fig. 3:**
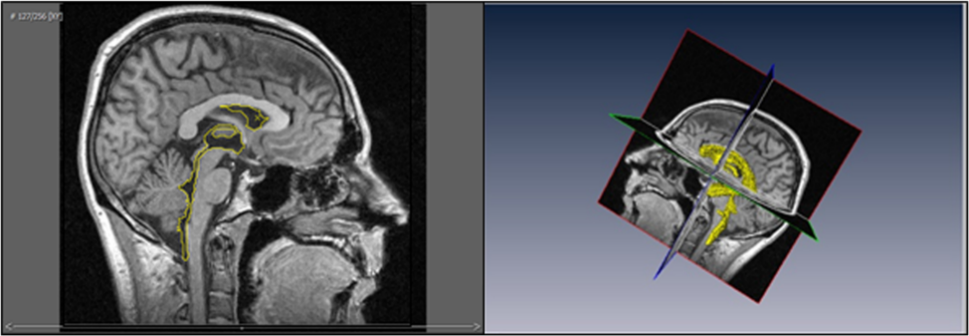
Split screen in Amira 5.4.5 showing both the 2D plane from the dataset (left) and 3D model (right), allowing easier segmentation in unclear areas

**Fig. 4:**
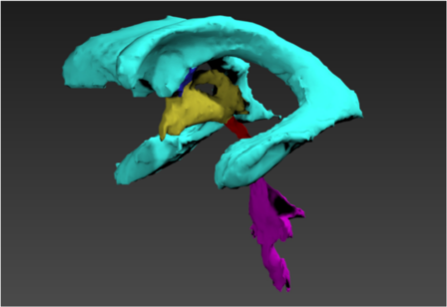
The five selected parts of the ventricular system in Autodesk 3DS Max as separate models

The 3D ventricular system model was manually segmented in Amira 5.4.5. Fig. 3 shows the split screen of Amira 5.4.5 during the segmentation process performed on the dataset 2D slices over the x, y and z axes. Fig. 4 shows the sections of the segmented ventricular model in Autodesk 3DS Max, after smoothing in Sculptris.

### 3D models

**Fig. 5:**
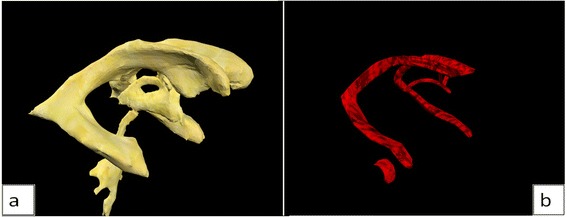
This shows (**a**) the ventricular system model textured with bitmap, and (**b**) the choroid plexus model, textured using bitmap and bump map

The ventricular system model was textured using a bitmap. Models of the choroid plexus were created manually in Autodesk 3DS Max, based on choroid plexus location reported in the literature, then textured using both a bitmap and bump map to generate the sensation of relief onto their surface (Fig. 5).

### Animations

#### Introduction animation

**Fig. 6:**
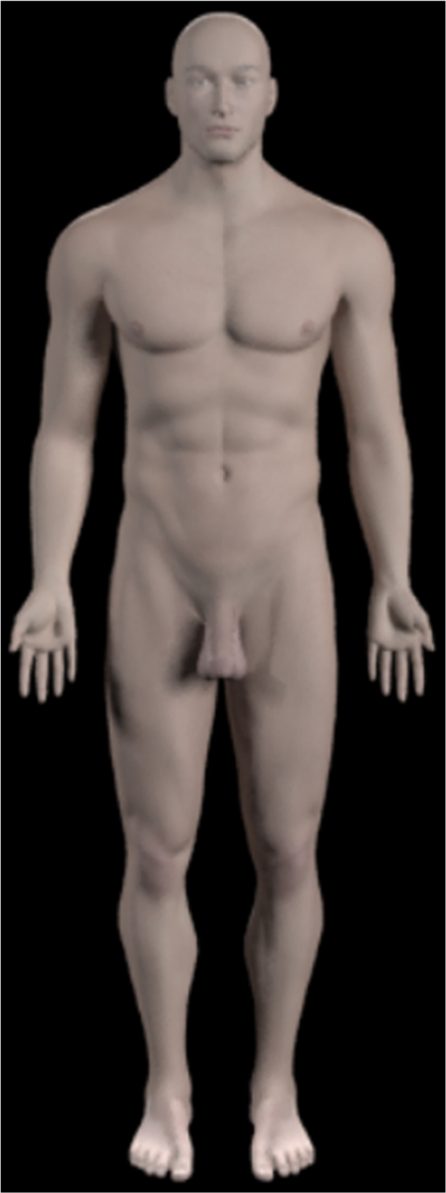
Still image from the start of the introduction animation showing the whole body

**Fig. 7:**
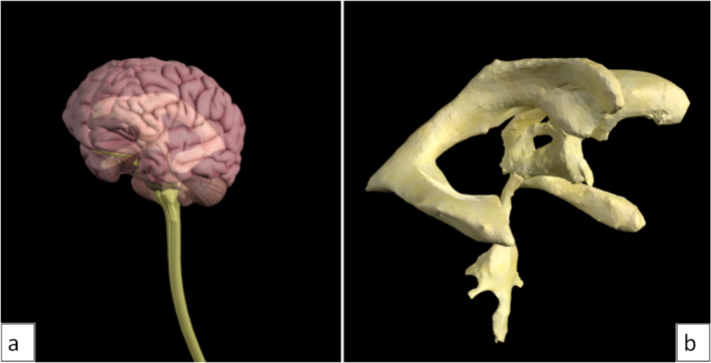
Still images from the introduction animation showing (**a**) the brain and spinal cord models fading out to reveal (**b**) the ventricular system model

The introduction animation began with a front view of the whole body (using the Zygote Body skin model; Fig. 6). As the models rotate and the camera zooms in, the skin model fades out revealing the Zygote Body brain and spinal cord models and the 3D ventricular system model (Fig. 7).

#### CSF flow animation

**Fig. 8:**
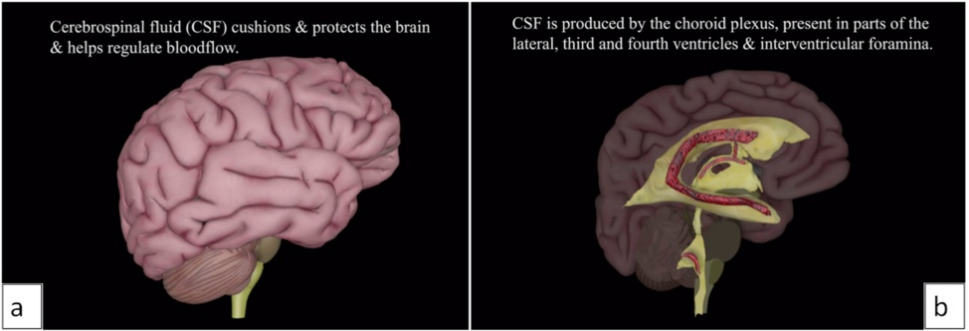
Still images from the CSF flow animation showing (**a**) the brain and spinal cord models. The right hemisphere of the brain then fades out, along with part of the ventricular system model to show (**b**) the 3D ventricular system model and choroid plexus

The CSF flow animation starts with a side view of the Zygote Body brain and spinal cord models (Fig. 8). The hemisphere nearest the camera (right hemisphere) fades out to show the ventricular system model. The left hemisphere of the brain fades to 30 % opacity and the part of the ventricular model nearest the camera fades to 0 % opacity, revealing the choroid plexus model.

**Fig. 9:**
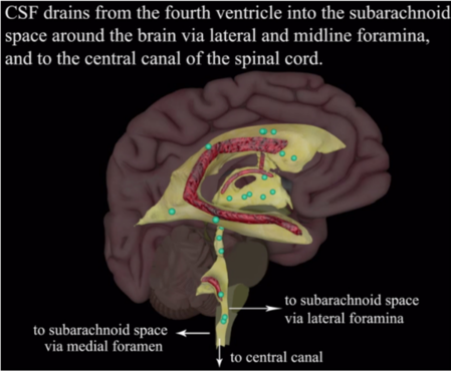
Still image from the CSF flow animation showing small light blue spheres representing CSF. This demonstrates the flow of CSF through the ventricles with arrows and text indicating how the CSF leaves the fourth ventricle

Small light blue spheres representing cerebrospinal fluid appear and travel along paths to highlight the flow of CSF through the ventricles (Fig. 9). Arrows and text, added in Adobe After Effects CC appear indicating the routes by which CSF leaves the fourth ventricle and enters subarachnoid space and the central canal of the spinal cord.

### Interactive application

#### Interactive tutorial

In the interactive model tutorial, the user can adjust the transparency of the skin, skull and brain (BodyParts3D models) using horizontal sliders, enabling the spatial relationship between the ventricular system and surrounding structures to be explored. The choroid plexus can be viewed by adjusting the transparency of the ventricular system. When the mouse passes over an area of the ventricular system that area turns red and its name appears at the top of the screen.

**Fig. 10:**
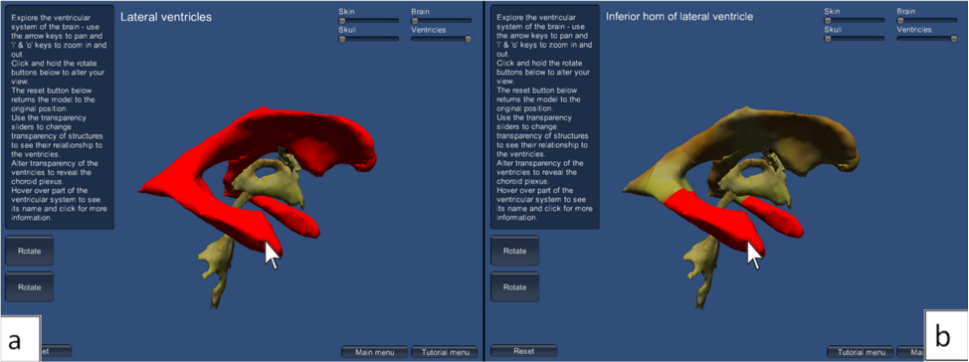
These show the scene information box and rotate menu buttons in the 3D interactive model tutorial. When the mouse passes over a section of ventricular system, it turns red and its name appears above, as seen in (**a**) easy and (**b**) hard tutorial levels

**Fig. 11:**
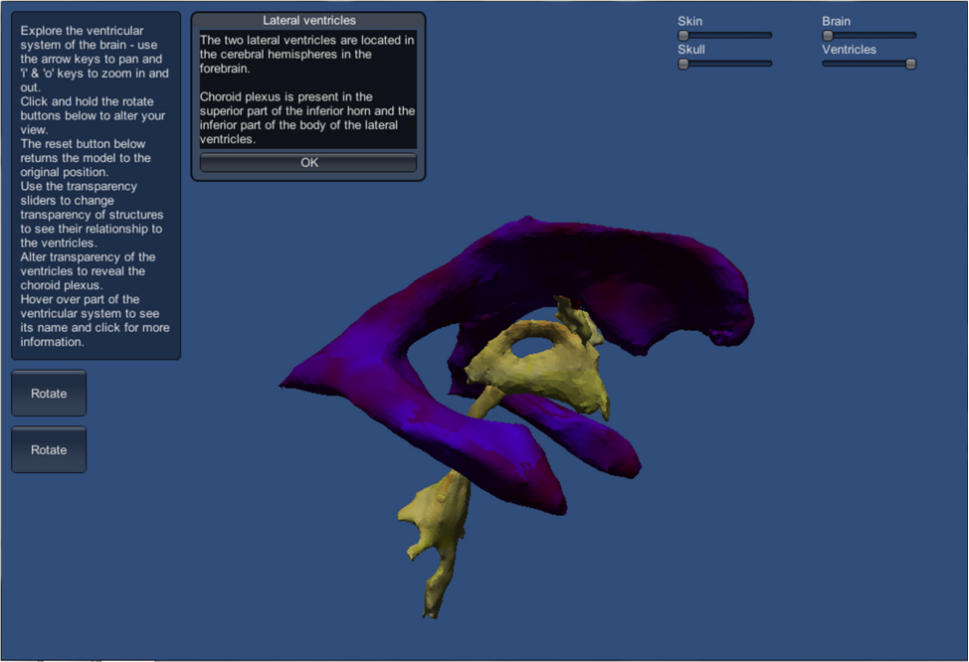
This demonstrates when an area of the ventricular system model is clicked, it turns blue and a pop-up window appears with more information

Figure 10 shows the difference between the easy and hard tutorial scenes – the hard model tutorial is more detailed as it contains the subdivided lateral, third and fourth ventricles, allowing smaller sections of the model to be selected. The figure shows buttons on the left of the screen which allow rotation of the model around the x- and y-axes, allowing it to be seen from all angles. The user can also zoom in and out and pan in two axes. The model tutorial scene also contains a button to reset the orientation of the model and buttons linking back to the tutorial menu and the main menu. When an area of the ventricular system model is clicked, it turns blue and a pop-up window appears with information about that area of the model (Fig. 11).

#### CSF Flow tutorial

In the CSF flow tutorial, the CSF flow animation can be launched and paused by clicking a button at the left of the screen. The background music can also be played and paused using this button. At the bottom left of the screen, another button contains a pop-up window providing information about CSF and the choroid plexus. Additional buttons at the bottom right of the screen link to the tutorial menu and the main menu.

#### Labelling challenge

**Fig. 12:**
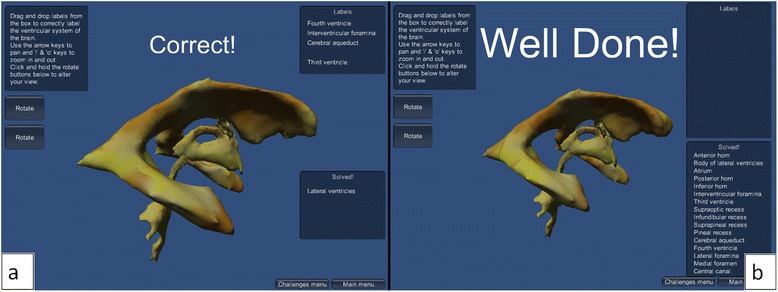
This shows the labelling challenge scenes with (**a**) one label correctly assigned, giving a “Correct!” augmented feedback (visual and auditory), in the easy difficulty level, and (**b**) all labels correctly assigned, giving “Well done!” and audio augmented feedback, in the hard level

The labelling challenge, in the test-your-knowledge section of the interactive application, has a labels box containing the labels to be dragged and dropped onto the model. There are also buttons within the level to rotate the model to allow it to be seen from all angles. Once a label has been dragged onto the correct area of the ventricular system, the label "jumps" to the “solved” box at the side, and visual and auditory augmented feedback are provided: visual textual feedback (“Correct!”) and a sound, both providing positive reinforcement. If the label is dragged onto the wrong section of the ventricular system model, augmented feedback consists of: “Try again” textual indicator and a sound with negative connotation are provided. When all labels have been correctly assigned, terminal augmented feedback is provided as “Well done!” text appears and a third sound is played (Fig. 12).

#### Multiple choice quiz

**Fig. 13:**
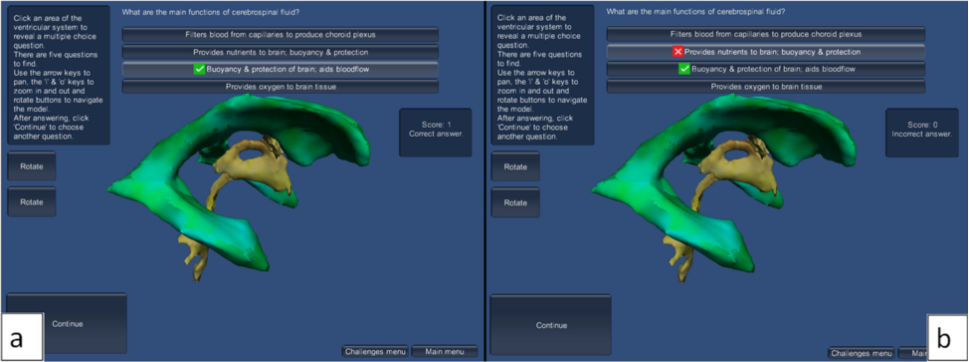
This demonstrates when (**a**) answering correctly and (**b**) answering incorrectly

In the multiple choice quiz, rotate buttons on the left adjust the model’s position and buttons at the bottom-right return the user to the test-your-knowledge menu and to the main menu. When the mouse is passed over an area of the ventricular system model it turns red, allowing the user to easily find any remaining questions. On clicking a section of the ventricular model, a multiple choice question pops up, and the selected area of the model remains red. After answering the question, that part of the model turns blue-green, to make it easy to see which areas still have questions to be answered. When providing a correct answer, a green tick appears beside the statement, and audio feedback is played. Score is increased by one point and the message “Correct answer” is displayed. When providing a wrong answer, a red cross appears beside the chosen wrong statement and a green tick beside the correct one, allowing the user to learn from their mistakes. A different sound is played and the text saying “Incorrect answer” is shown. To move to the next question, the user clicks the “Continue” button (Fig. 13).

When the quiz is completed, terminal augmented feedback is given in the form of a final audio cue and the final score is given along with either a congratulatory message or a message encouraging the user to revisit the tutorials and attempt the quiz again, depending on the score achieved.

## Discussion

The development and creation of 3D reconstructions of anatomical structures from medical scanning data have only been made possible recently by advances in medical imaging techniques. The combined use of these medical imaging datasets and recently developed computer software allows the creation of anatomically accurate 3D models from 2D medical imaging datasets, improving anatomical education and understanding.

Some areas of anatomical teaching can be difficult to explain, difficult to see in anatomical specimens, and difficult to imagine and understand, especially the spatial relationship between multiple structures [27]. The use of anatomically accurate 3D models in computer-assisted learning can greatly aid learning and perception in these complex areas when compared with individuals not exposed to the 3D model [32, 33], especially when the model has been created using actual medical data, which avoids a potentially misleading “artist’s impression”. The use of computer-aided learning as a teaching aid is gaining popularity in the anatomical curriculum and is proving valuable for the learning of more complex areas of anatomy, especially when using 3D interactive models generated from real medical data. It can have beneficial effects with regard to awareness of shape and spatial interactions between different anatomical structures, especially when used in concert with other teaching methods such as cadaveric dissection [2, 13]. Those with a preference for a more visual learning style may be more engaged in these more integrated teaching methods, rather than traditional textbook-based learning, although there has been shown to be little correlation between positive feedback about static versus dynamic visualisations and learning outcomes [18]. The interactive application created during this study incorporates both textual information and interactive visual information in the form of animations and user manipulation of the model itself, and should therefore appeal to individuals with different learning preferences.

The ventricular system of the brain can be difficult to imagine and understand as it is commonly only seen in 2D on medical imaging scans or cadaveric prosections which fail to give an impression of the 3D structure of the ventricular system. Casts of the ventricles give an improved understanding of their general shape but do not usually show the position of the ventricular system within the brain or give any idea of the production or flow of CSF, both of which are very important points to consider in the study of the ventricular system. The ventricular system of the brain benefits from computer-based learning due to its complexity and the difficulty with which it is viewed. We set out to develop a workflow pipeline to create anatomical datasets and interactive educational material using widely available software packages. This study has shown the ease at which set protocols have been established in the creation of this interactive educational material. The method created in this study, of animations being shown within the interactive application (for example illustrating the flow of CSF around the ventricles) demonstrates great interactivity not previously created to our knowledge. 3D computer models can offer an element of interaction that cannot be achieved through physical casts of the ventricles, or through medical imaging data, which aids understanding of the anatomical shape and physiological function of the ventricular system of the brain while also being more stimulating to the user [9–11]. In addition to this, our workflow presented here also incorporates interactive quizzes, and the ease at which this can be done to build on and enhance that currently available [23].

### Discussion of results

In this study, we have presented a workflow pipeline through which we have processed anatomical dataset in order to generate an accurate 3D model of the cerebral ventricular system. 3D rendering and game engine platforms were used to provide realistic texturing to the model, and to generate several animations and interactive applications.

With this established workflow created here, this application has the potential to be used for those students studying the head and neck region of the body either as a classroom-based exercise or in student-directed learning. It has many additional benefits which could be attractive to the student users as it contains a tutorial section with the 3D ventricular reconstruction, is interactive (pan, zoom and rotate facilities enabling a 360° view of the ventricular system), and presents information about the various areas of the ventricular system available at a click of the mouse. The transparency of the ventricular system model and of the skin, skull and brain from BodyParts3D models can also be adjusted to enhance the understanding of the spatial relations between the various elements to be seen. The tutorial section also incorporates the two animations: one as an introduction to the ventricular 3D model, starting with the whole body and zooming in to the ventricular system to give the user an idea of where in the body it is located; and the second animation to provide a simulation of CSF flow through the ventricular system of the brain, accompanied by explanatory text and a pop-up window containing more information about the choroid plexus and CSF production. Two difficulty levels are available for the interactive 3D model (easy and hard) which vary in the amount of detail given. With a test-your-knowledge section using drag-and-drop and a multiple-choice quiz, it provides not just knowledge transfer, but self-testing facilities. This provides the user with a unique way to interact with educational material developed through our workflow pattern which we have created. The interactive application can be played on both Microsoft® Windows and Mac OS X computing platforms as a standalone programme and can also be hosted online, using Unity Web Player, for wider access. Hopefully this workflow pattern and the incorporation of a variety of widely available software packages will enable other users and educationalists to follow this in the creation of datasets and self-assessment tools for a variety of body regions ideal for a wide variety of student group users.

### Limitations

Despite the presented development of a potentially useful educational tool, several limitations in the workflow we have presented remain. The use of manual segmentation tools meant that this model is not easily reproducible. Using a more semi-automated or automated approach to the segmentation process could help to overcome this issue.

Given more time, a voiceover could be recorded and included in the CSF flow animation as an alternative to background music. This may enhance the learning experience as individuals have different taste in music and some find background noise difficult to work with. Also, by including a voiceover the user has an alternative to reading the text, which some individuals may prefer, with studies suggesting that individuals learn better from animation and narration than from animation and onscreen text [19, 34]. A “mute” button could also be included, so the user can decide if they wish a voiceover or not.

Given more development, the labelling challenge could be improved by enabling the “Solved!” labels to remain with the correct area of the model. This would result in a labelled interactive model which the user could then study in detail, having been labelled by them.

In addition, the question base for the multiple-choice quiz is limited. With further development this could be expanded and could incorporate random selection of the questions so the quiz could be repeated with different styles and types of questions.

### Future study

The model and learning applications have been developed in the first instance to test the viability of the creation of the animations, tutorials and interactivity. We have indeed shown this to be achievable in the creation of a training and educational application using modern technologies. Validation of its use for undergraduate students looking to gain knowledge of the neurological system would be needed to ensure it was user-friendly and effective. This would have to involve comparative studies with those using the package created here, and those not using it, and its effectiveness, or otherwise recorded, perhaps by assessment of knowledge gained.

As it stands, the application files can be loaded onto computers or shared over the internet without the need for specialist software, but there is the potential for this application to be developed to run fully online, using Unity Web Player, without the need for download which would improve student’s access to the application. It could also be considered for smartphones and tablets, allowing students to access it and learn “on-the-go”. Furthermore, tactile interface through tablets may strongly improve the interaction that users have with the 3D model. This may lead to an increase in the user’s involvement and thus enhance learning.

This learning application could be developed further to enable stereoscopic display in order to enhance the perception of depth in the model, for a greater understanding of the spatial relationships between the separate structures and increase the user’s immersion, enhancing learning.

The application could be enhanced by adding some examples of pathologies affecting the ventricular system of the brain, for example obstructive hydrocephalus, and could include surgical simulations of techniques used to drain the excess CSF such as cerebral shunt or endoscopic third ventriculostomy, either as a teaching animation or as an interactive task.

In order to expand the usefulness of this interactive teaching application, areas of the brain could also be segmented and added as a separate section of the application, with tutorials and learning challenges to undertake. In an interactive application dealing with the entire brain, it may be useful for the user to be able to turn off some structures at will, allowing other, hidden structures to be viewed.

## Conclusion

The aim of this study was to establish a workflow pattern using widely available software packages in the creation of a tool which could be used for a variety of groups of students and professionals requiring knowledge of the cerebral ventricles and flow of CSF. We have successfully constructed a fully interactive educational and training package for this use. Further validation of this would certainly be needed as to its educational benefit. However, we now have created a way for future educators, specialists and end users to become engaged with the development of these methods in the creation of tailor made packages through our workflow presented here.
